# High Intensity Interval Training (HIIT) Induces Specific Changes in Respiration and Electron Leakage in the Mitochondria of Different Rat Skeletal Muscles

**DOI:** 10.1371/journal.pone.0131766

**Published:** 2015-06-29

**Authors:** Dionizio Ramos-Filho, Gustavo Chicaybam, Eduardo de-Souza-Ferreira, Camila Guerra Martinez, Eleonora Kurtenbach, Gustavo Casimiro-Lopes, Antonio Galina

**Affiliations:** 1 Laboratory of Bioenergetics and Mitochondrial Physiology-Institute of Medical Biochemistry Leopoldo de Meis-Federal University of Rio de Janeiro, UFRJ, Rio de Janeiro, Rio de Janeiro, Brazil; 2 Institute of Physical Education and Sports-State University of Rio de Janeiro, UERJ, Rio de Janeiro, Rio de Janeiro, Brazil; 3 Institute of Biophysics Carlos Chagas Filho, Federal University of Rio de Janeiro UFRJ, Rio de Janeiro, Rio de Janeiro, Brazil; Tohoku University, JAPAN

## Abstract

High intensity interval training (HIIT) is characterized by vigorous exercise with short rest intervals. Hydrogen peroxide (H_2_O_2_) plays a key role in muscle adaptation. This study aimed to evaluate whether HIIT promotes similar H_2_O_2_ formation via O_2_ consumption (electron leakage) in three skeletal muscles with different twitch characteristics. Rats were assigned to two groups: sedentary (n=10) and HIIT (n=10, swimming training). We collected the tibialis anterior (TA-fast), gastrocnemius (GAST-fast/slow) and soleus (SOL-slow) muscles. The fibers were analyzed for mitochondrial respiration, H_2_O_2_ production and citrate synthase (CS) activity. A multi-substrate (glycerol phosphate (G3P), pyruvate, malate, glutamate and succinate) approach was used to analyze the mitochondria in permeabilized fibers. Compared to the control group, oxygen flow coupled to ATP synthesis, complex I and complex II was higher in the TA of the HIIT group by 1.5-, 3.0- and 2.7-fold, respectively. In contrast, oxygen consumed by mitochondrial glycerol phosphate dehydrogenase (mGPdH) was 30% lower. Surprisingly, the oxygen flow coupled to ATP synthesis was 42% lower after HIIT in the SOL. Moreover, oxygen flow coupled to ATP synthesis and complex II was higher by 1.4- and 2.7-fold in the GAST of the HIIT group. After HIIT, CS activity increased 1.3-fold in the TA, and H_2_O_2_ production was 1.3-fold higher in the TA at sites containing mGPdH. No significant differences in H_2_O_2_ production were detected in the SOL. Surprisingly, HIIT increased H_2_O_2 _production in the GAST via complex II, phosphorylation, oligomycin and antimycin by 1.6-, 1.8-, 2.2-, and 2.2-fold, respectively. Electron leakage was 3.3-fold higher in the TA with G3P and 1.8-fold higher in the GAST with multiple substrates. Unexpectedly, the HIIT protocol induced different respiration and electron leakage responses in different types of muscle.

## Introduction

The World Health Organization has stated that physical inactivity is the fourth leading risk factor for global mortality (6% of deaths worldwide), which is equivalent to approximately 3.2 million deaths per year [1]. The American College of Sports Medicine recommends at least 20 minutes a day of vigorous exercise three times per week [2]. High intensity interval training (HIIT) is characterized by periods of high intensity exercise combined with short rest intervals, resulting in aerobic-like effects. In addition, HIIT protocols shorten the total time spent on physical activity while producing the same work load [3, 4]. This training strategy is currently applied in humans as an alternative exercise intervention in different disease conditions, such as heart failure, hypertension, type II diabetes, obesity and chronic obstructive pulmonary disease [5–10].

In rat muscle, HIIT protocols favor high citrate synthase (CS) and hydroxyacyl-CoA dehydrogenase (HAD) activities, mitochondrial gene expression and mitochondrial biogenesis [3, 11–13]. Rats with a high running capacity have low levels of oxidative stress, suggesting an increase in antioxidant defenses and/or low production of reactive oxygen species (ROS) by the mitochondrial electron transport system (ETS) [14]. In previous work [15], we demonstrated that HIIT resulted in high endurance and low mitochondrial glycerol phosphate dehydrogenase (mGPdH) activity. This enzyme is part of the glycerol phosphate shuttle in the inner mitochondrial membrane, which is the site of the third highest rate of mitochondrial superoxide (O_2_^−•^) production [16]. Our previous study indicated that HIIT decreases mGPdH activity, suggesting that HIIT prevents ROS production. In fact, decreased mGPdH activity has been previously demonstrated] 15], but the mitochondrial ROS production rate was not evaluated, and the effects of HIIT protocols on hydrogen peroxide (H_2_O_2_) generation are unknown.

During mitochondrial respiration, a small portion of the electrons leak out from the ETS, primarily through complexes I and III, and form O_2_^•−^ and other ROS [17, 18]. These electron leaks may be an indicator of the mitochondrial redox status and could be involved in signaling for muscle remodeling and atrophy [19]. Aerobic endurance training reduces mitochondrial electron leakage in the gastrocnemius (GAST) muscle in the presence of malate plus glutamate or succinate (Succ) [20]. These substrates favor ROS formation at complexes I and III. However, the effect of HIIT protocols on the mitochondrial generation of ROS, which is sustained by glycerol phosphate (G3P), fatty acids or mixed substrates, is not fully understood. Endurance training increases the oxidative capacity of skeletal muscle. Thus, the aim of this study was to evaluate whether HIIT would alter mitochondrial respiration and H_2_O_2_ production in different types of permeabilized skeletal muscle fibers (tibialis anterior (TA), fast (F); GAST, fast/slow (F/S); and soleus (SOL), slow (S) in a similar manner and to determine the fraction of H_2_O_2_ formed from O_2_ consumption after HIIT (electron leakage).

## Methods

### Animals

Twenty 90-day-old male Wistar rats were housed with a 12-h dark/light cycle and ad libitum access to food and water. The rats were matched by weight and randomized into two groups: sedentary (SED; n = 10) and HIIT (n = 10). The experimental protocol was approved by the Animal Ethics Committee, Federal University of Rio de Janeiro (Permission Number: 012000.001568/2013-87). Three types of skeletal muscle from the same hindlimbs were analyzed: TA fast fibers (92% type IIb fast twitch fibers), GAST mixed fibers (approximately 50% type I slow twitch and type II fast twitch fibers) and SOL slow fibers (84% type I slow twitch fibers) [21]. These three muscles were chosen to analyze mitochondrial physiological adaptations after the HIIT swimming protocol because the TA and GAST are very active during swimming; indeed, glycogen levels decrease by 75% and 50% in the TA and GAST, respectively [22, 23]. Conversely, glycogen levels do not change in the SOL [22]. The SOL was therefore used as the negative swimming control.

### Exercise protocol

Rats were exercised by swimming as described by Terada et al. [3]. The HIIT consisted of fourteen bouts of 20-sec swimming periods, each followed by a 10-sec rest. This protocol was administered three times a week on alternating days. The initial load was 9% of the body weight, and this was incrementally increased by 1% of the body weight each week. The SED controls were untrained. Training adaptation was evaluated at the end of 6 weeks, when the controls and HIIT rats were assessed in an acute test of swimming endurance in which a load of 14% of the body weight was applied [15].

### Skeletal muscle sampling and tissue collection

The rats were decapitated 48 h after the last exercise. The body weight and visceral fat mass were measured, and the body fat percentage was calculated for each animal. Skeletal muscles (TA, GAST and SOL) were excised and placed at 4°C until analysis. Skeletal muscle preparation. This technique has been described in detail by Anderson and Neufer [24] and Pesta and Gnaiger [25]. After dissection, the muscle samples were placed in ice-cold (4°C) BIOPS buffer (~3 mg wet weight per fiber bundle). The fiber bundles were treated with saponin (50 μg/ml) for 30 min as previously described [25]. All the preparations were performed at 4°C, and the samples were stored at 4°C; 2 h after permeabilization, the fibers were used for respirometry and H_2_O_2_ production experiments. At the end of the experiments, skeletal muscle biopsies were stored at −70°C for further analysis of CS activity and western blots.

### Respiratory protocol

Respiration measurements were performed in 2 ml of mitochondrial respiration medium 05 (MiR05) for each fiber bundle. O_2_ consumption was measured using the high-resolution Oxygraph-2k system (Oroboros, Innsbruck, Austria) [25, 26]. The results were normalized to the wet weight or CS activity of the permeabilized fiber bundles. All the experiments were performed at 37°C in a 2-ml chamber. Oxygen flux induced by the addition of cytochrome c (Cyt-c) increased no more than 10%, confirming the viability of the preparations during the experiments [26]. Multi-substrate titrations. This titration protocol was modified from previous protocols that have been described in detail [25]. All the titrations were performed in series as described below. The multi-substrate titration consisted of the sequential addition of G3P (10 mM); pyruvate, malate, and glutamate (PMG; 10, 10, and 20 mM, respectively); Succ (10 mM); adenosine diphosphate (ADP, 2.5 mM); Cyt-c (10 μM); oligomycin (Oligo, 2 μg/ml); carbonyl cyanide p-(trifluoromethoxy) phenylhydrazone (FCCP, 1 μM); rotenone (ROT, 0.5 μM); malonate (MALO, 10 mM); and cyanide (KCN, 5 mM). Evaluation of fatty acid oxidation: A second titration protocol was performed sequentially with G3P (10 mM; non-p-state) and ADP (2.5 mM; p-state). A third titration protocol involved palmitoyl-carnitine (PALM, 75 μM; non-p-state) and ADP (2.5 mM; p-state).

### Citrate synthase enzyme activity

CS activity was measured in all three muscles as described previously [27] with minor modifications. TA, GAST and SOL muscle samples were homogenized in lysis buffer (50 mM sodium phosphate, pH 7.4, 10% glycerol, 1% octal phenol ethoxylate, 10 mM sodium orthovanadate, 10 mM NaF, and 10 mM sodium pyrophosphate) supplemented with a protease inhibitor cocktail (P8340, Sigma-Aldrich) using an Ultra Turrax T25 (IKA, Staufen Werke, Germany). After 30 min on ice, the tissue lysates were centrifuged (13,000 x g for 20 min at 4°C), and the resulting supernatants were collected.

The reaction medium contained 20 mM Tris-HCl, pH 8, 0.3 mM 5,5’-dithio-bis(2-nitrobenzoic acid) (DTNB), 0.48 mM acetyl coenzyme A (AcCoA), and 5 μg/ml protein. The medium was incubated for 5 min with continuous shaking at 37°C, and the reaction was initiated by the addition of 5 mM oxaloacetate. The reactions were performed in a 96-well plate, and the results were read at 420 nm in a Victor-Perkin Elmer plate reader. The specific activity was calculated based on a curve of CoA in the presence of 0.3 mM DTNB and was expressed as μmol citrate.min^-1^.mg^-1^ protein.

### H_2_O_2_ production in permeabilized fibers

Mitochondrial H_2_O_2_ release was determined by measuring the fluorescence of the Amplex Red probe (Life Technologies SAS, Brazil) using a spectrofluorimeter (Varian Cary Eclipse; Agilent Technologies, Santa Clara, CA) as described previously (24). The reaction was initiated by the addition of a bundle of fibers that were permeabilized with 2 ml of MiR05 solution containing 5 μM Amplex Red and 4 U/ml horseradish peroxidase. The following solutions were added sequentially in the multi-titration experiment: G3P (10 mM), PMG (5:5:10 mM), Succ (10 mM), ADP (2.5 mM), Oligo (2 μg/ml), FCCP (0.5 μM), and the complex III blocker antimycin A (Ant A; 2.5 μM). The second and third titration protocols described above were performed sequentially with the same additives as in the first titration. The second titration used only one substrate, G3P (10 mM), along with ADP (2.5 mM), Oligo (2 μg/ml), FCCP (0.5 μM), and Ant A (2.5 μM), whereas the third titration used only PALM (75 μM), ADP (2.5 mM), Oligo (2 μg/ml), FCCP (0.5 μM), and Ant A (2.5 μM). The fourth and fifth titrations used the following sequential titration with a different initial substrate in the TA and GAST skeletal muscles: for the fourth titration, G3P (10 mM), ROT (0.5 μM), MALO (10 mM) and Ant A (2.5 μM); for the fifth titration, PALM (75 μM), ROT (0.5 μM), MALO (10 mM) and Ant A (2.5 μM). H_2_O_2_ was measured, and the results were expressed per milligram of wet weight and normalized to CS activity when necessary. The specific rate of H_2_O_2_ production was determined using linear regression curve fitting with Origin 8.0.

### Electron leakage determination

We calculated the fraction of electrons that leaked out of the respiratory chain by dividing the rate of H_2_O_2_ formation by the rate of O_2_ consumption [20, 28]. To calculate electron leakage, these 2 measurements were expressed using the same units and normalized to CS activity. The electron leakage related to a specific substrate was calculated as the ratio of H_2_O_2_ formed by a specific substrate (for example, G3P) or by a multi-substrate regimen (for example, G3P/PMG/Succ) to oxygen consumption with that substrate or substrate regimen.

### Western blot

Samples were taken from each muscle and macerated in HEPES buffer (50 mM HEPES, 10 mM EDTA, 1 mM MgCl_2_, and 0.1% Triton X-100) using the Ultra-Turrax homogenizer [29]. The samples were weighed and centrifuged to remove debris. The supernatant was collected, and 20 ug of each protein sample was resolved by 4–20% SDS-PAGE (Mini-Protean TGX 4–20%, BioRad) at 50 V and transferred to a PVDF membrane (Trans-Blot Transfer Turbo Pack, BioRad) according to the product specifications for the Trans-Blot Transfer System (BioRad). After transfer, the membranes were blocked with 5% BSA in 1X TBS-T (10 mM Tris, pH 8.0, 150 mM NaCl, and 0.5% Tween 20) for 2 h and incubated overnight at 4°C with gentle agitation with the following primary antibodies: Anti-Rt / MS Total OXPHOS (Invitrogen; 1:2500 dilution) and GAPDH (Santa Cruz Biotechnology; 1:20,000 dilution). The specific HRP-conjugated secondary antibody was incubated with the membranes for 2 h at room temperature. After this procedure, the membranes were developed using the ECL Plus Chemiluminescent Western Blotting Detection System (GE Healthcare Life Sciences).

### Statistical analysis

Comparisons were performed using an unpaired t-test. The data are presented as the mean ± standard error, and p<0.05 was defined as significant. To analyze and quantify the rate of H_2_O_2_ production, we analyzed the linear regressions from the stationary flows after the addition of different substrates or inhibitors using the Origin graphic and statistics analysis program to calculate the slope of the curve. Then, the picomolar concentration of H_2_O_2_ was calculated using the slope of the curve.

## Results

### Effects of training on performance, body weight and % visceral fat mass

The efficacy of HIIT was confirmed based on the more than 2.0-fold increase in the swimming duration of the HIIT rats compared to the SED controls at the end of the training period (p<0.05). In addition, the HIIT rats gained less body weight (7%; p<0.05) than the SED controls, and the % visceral fat mass of the HIIT rats was proportionally lower (22%; p<0.05). The masses of the three muscles in the HIIT group were not different than those in the SED group (Table 1).

**Table 1:** Maximal Endurance Test (s), body weight, visceral fat, visceral fat percentage, and muscle weight in rats subjected to chronic high-intensity training (HIIT) over 6 weeks.

	SED	HIIT	P values
**Swimming duration (s)**	**174 ± 34 (9)**	**349 ± 26** * **(10)**	**p<0.01**
**Body weight (g)**	**378 ± 9.5 (9)**	**352 ± 8.0** * **(10)**	**p<0.05**
**Visceral fat (g)**	**6.8 ± 0.60 (9)**	**5.3 ± 0.63** * **(10)**	**p<0.05**
**Visceral fat (%)**	**1.8 ± 0.1 (9)**	**1.4 ± 0.2** * **(10)**	**p<0.05**
**Tibialis anterior**	**0.591 ± 0.13 (9)**	**0.605 ± 0.09 (10)**	**NS**
**Gastrocnemius**	**1.79 ± 0.15 (9)**	**1.78± 0.12 (10)**	**NS**
**Soleus**	**0.170 ± 0.04 (9)**	**0.173 ± 0.05 (10)**	**NS**

The number of animals is shown in parentheses. The data are expressed as the mean ± standard error. NS: not statistically significant.

* p<0.05 vs controls.

### HIIT Induces differential oxygen consumption flux in distinct skeletal muscles: Data from multiple substrates (glycerol phosphate; pyruvate, malate, and glutamate; and succinate) normalized to wet weight (mg)

Previous work [15] has shown that mGPdH activity decreases by more than 80% in the TA skeletal muscle after HIIT. However, the effect of HIIT on mitochondrial respiration, which is supported by mGPdH activity and multiple substrates, was not measured in different muscles. To clarify this point and confirm the kinetics of mGPdH activity, a high-resolution respirometry experiment was performed in three types of muscle with different demands during swimming work.

**Fig 1:**
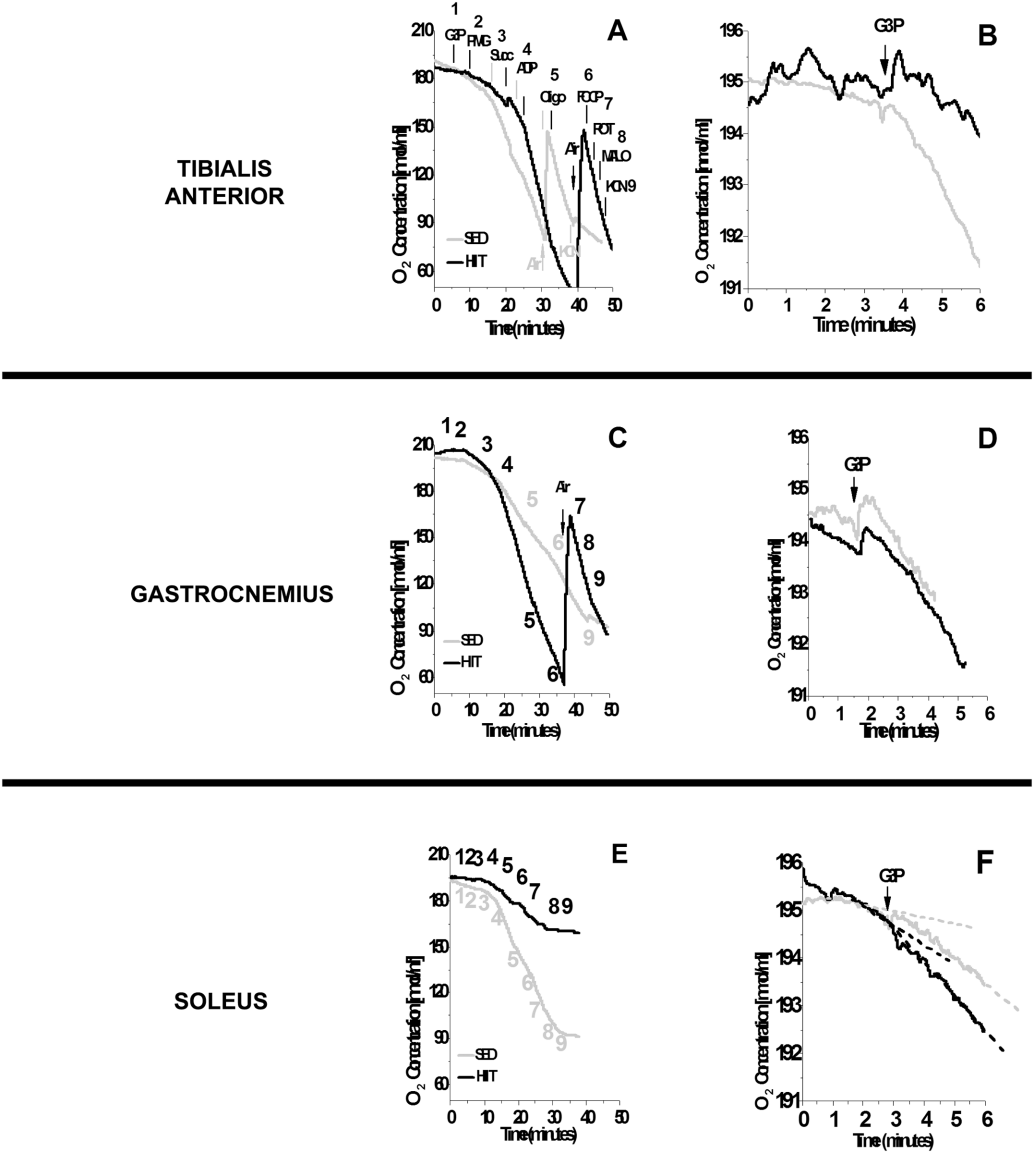
Oxygen depletion by different skeletal muscles using multiple substrates (A, C and E) or glycerol phosphate only (B, D and F). (A and B) Tibialis anterior. (C and D) Gastrocnemius. (E and F) Soleus. 1, G3P (10 mM); 2, PMG (5:5:10 mM); 3, Succ (10 mM); 4, ADP (2.5 mM); 5, Oligo (2 μg/ml); 6, FCCP (1 μM); 7, ROT (1.0 μM); 8, MALO (10 mM); 9, KCN (5 mM). Black (HIIT); gray (SED).

**Fig 2:**
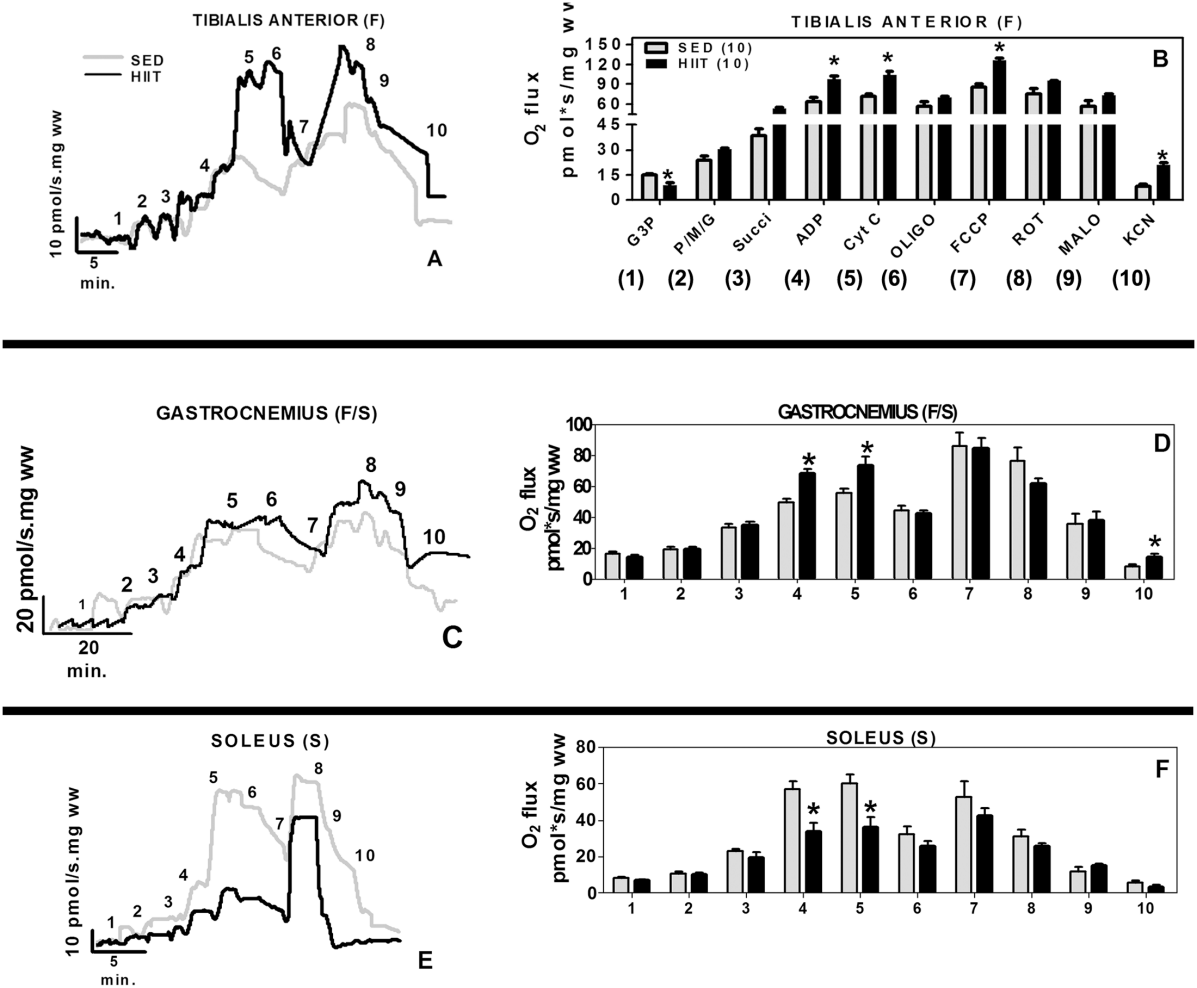
Oxygen flux in high-resolution respirometry experiments per fiber mass. (A and B) Tibialis Anterior. (C and D) Gastrocnemius. (E and F) Soleus. 1, G3P (10 mM); 2, PMG (5:5:10 mM); 3, Succ (10 mM); 4, ADP (2.5 mM); 5, CYT C (10 μM); 6, Oligo (2 μg/ml); 7, FCCP (1 μM); 8, ROT (1.0 μM); 9, MALO (10 mM); 10, KCN (5 mM). Black (HIIT); grey (SED). The data are expressed as the mean ± standard error. *p<0.05, HIIT vs SED. The number of individual experiments is in parentheses.

Fig 1 illustrates the oxygen depletion rates in the TA (F), GAST (F/S) and SOL (S) after the sequential addition of a multi-substrate regimen (A, C and E), and detailed data are presented specifically for mGPdH activation by G3P (B, D and F). The HIIT protocol promoted a higher rate of oxygen consumption in the presence of G3P/PMG/Succ and ADP in TA (F) and GAST (F/S) fibers compared to the SED protocol (Fig 1A and 1C). Unexpectedly, there was a reduction in the rate of oxygen depletion in SOL (S) fibers derived from HIIT-trained rats compared to SED rats (Fig 1E). Interestingly, when G3P was added before the other substrates, there was less oxygen depletion in TA (F) fibers from HIIT-trained rats (Fig 1B). HIIT did not have an apparent effect in GAST (F/S) and SOL (S) fibers (Fig 1D and 1F). Thus, we analyzed additional fiber samples from both groups by high-resolution respirometry to confirm these results (Fig 2).

We observed differential effects of training on different muscles, such as the TA (F), GAST (F/S) and SOL (S) (Fig 2). The multi-substrate respirometry experiments showed that HIIT increased the respiratory capacity of permeabilized TA muscle fibers but only caused a small improvement in respiration in the GAST (Fig 2A and 2C, black traces). Unexpectedly, HIIT reduced the ADP-stimulated respiration in the SOL by more than 40% (Fig 2E, black trace). The HIIT protocol significantly changed the specific oxygen flux in these muscles (Fig 2B, 2D and 2F, black bars).

In the TA (F), the G3P-induced oxygen flux was reduced in the HIIT group by approximately 67%, but the ADP/Mg-induced oxygen flux was 1.5-fold higher than the control values (p<0.05) and was coupled to ATP synthesis (Table 2). The maximal uncoupled flux (ETS flux capacity) was increased by 1.3-fold relative to the control (p<0.05). In the GAST (F/S), the ADP/Mg-induced oxygen flux was 1.4-fold higher (p<0.05), and there were no significant changes in the maximal ETS flux capacity. The residual oxygen consumption (ROX) was 1.5-fold higher compared to that in the controls (p<0.05) (Table 2).

**Table 2:** Mitochondrial adaptations evaluated via O_2_ consumption induced by G3P/P/MG/Succ in tibialis anterior (F), gastrocnemius (F/S) and soleus (S) skeletal muscle fibers (pmol O_2_/s*mg wet weight).

	Tibialis anterior (F)		Gastro (F/S)		Soleus (S)	
Group	SED (8)	HIIT (10)	SED (8)	HIIT (7)	SED (7)	HIIT (6)
**Glycerol phosphate**	13.8 ± 2.4	9.3 ± 2.8 *	7.2 ± 1.9	7.8 ± 2.2	8.1 ± 1.1	7.2 ± 1.6
**Non-p-state multi**	38.5 ± 4.6	47.8 ± 3.1	33.3 ± 2.4	34.9 ± 2.1	23 ± 2.4	20 ± 2.3
**Maximal respiration (coupled)**	63.6 ± 18.5	98.4 ± 15.2 *	49.4 ± 2.6	68.3 ± 3.1 *	57.3 ± 8.5	33 ± 11 *
**Oligo respiration**	57.4 ± 16.3	69.8 ± 5.8	44 ± 2.1	42 ± 2.5	32.6 ± 2.3	25.6 ± 6.5
**ATP synthesis (O_2_ flow coupled to ATP–O_2_ flow with Oligo)**	6.2 ± 2.2	28.5 ± 9.4 *	5.2 ± 0.5	26.4 ± 0.5 *	24.7 ± 6.2	7.9 ± 4.5 *
**Coupled respiratory control (O_2_ flow ADP/O_2_ flow Oligo)**	1.1 ± 0.1	1.4 ± 0.1 *	1.3 ± 0.07	1.8 ± 0.1 *	1.8 ± 0.5	1.3 ±0.4 *
**Proton leak**	49.3 ± 7.4	19.5 ± 6.4 *	28.1 ± 4.2	19.6 ± 6.4	27.6 ± 2.7	22.3 ± 3.3
**Maximal respiration (uncoupled)**	85.8 ± 22.3	126.9 ± 10.2 *	85.8 ± 9.1	84.3 ± 7.5	53 ± 17	42 ± 10
**Uncoupled respiration control**	1.0 ±0.1	2.0 ± 0.2 *	1.5 ± 0.1	2.0 ± 0.1 *	1.5 ± 0.7	1.5 ± 0.1
**Reserve**	21.6 ± 7.8	45 ± 10.8	16.9 ± 4.4	19.1 ± 5.2	19.5 ± 4.1	16.5 ± 1.7
**Complex I**	9.8 ± 3.0	29.8 ± 7.7 *	14.4 ± 2.5	18.4 ± 3.4	31 ± 3.4	26 ± 1.7
**Complex II**	19 ± 2.3	51.9 ± 11.1 *	24.1 ± 2.3	41.8 ± 4.9 *	12.0 ± 2.2	15.1 ± 1.1
**mGPdH**	56.7 ± 9.5	18.7 ± 4.2 *	11.8 ± 3.8	8.2 ± 1.0	9.1 ± 0.9	11.8 ± 1.7
**ROX**	8.1 ± 4.7	20.3 ±2.6 *	9.3 ± 2.5	14.1 ± 2.9 *	5.7 ± 2.4	4.0 ± 2.5

The number of individual experiments is shown in parentheses. The data are expressed as the mean ± standard error.

* p<0.05 vs control.

In the SOL (S), the ADP/Mg-induced, respiration-coupled maximal oxygen flux was decreased by 42% in the HIIT group compared to the SED control group (p<0.05). The maximal uncoupled respiration was unchanged (Table 2).

Table 2 shows the specific changes in oxygen flux induced by substrates of the ETS. These values were derived from the use of ROT (complex I), MALO (complex II) and KCN to estimate the fraction of oxygen flux derived solely from mGPdH. This oxygen flux was reduced by approximately 70% (p<0.05). mGPdH-supported respiration was not affected by the HIIT protocol in either the GAST or SOL (Table 2).

Relative to the controls, the contribution of complex I to respiration increased (3.0-fold, p<0.05) in the TA muscle fiber preparations, but no differences were observed in the GAST or SOL fibers (Table 2). Complex II-supported respiration increased in TA fibers (2.7-fold, p<0.05) and in GAST fibers (1.7-fold, p<0.05), whereas no change was observed in SOL fibers (Table 2).

### Citrate synthase activity

**Fig 3:**
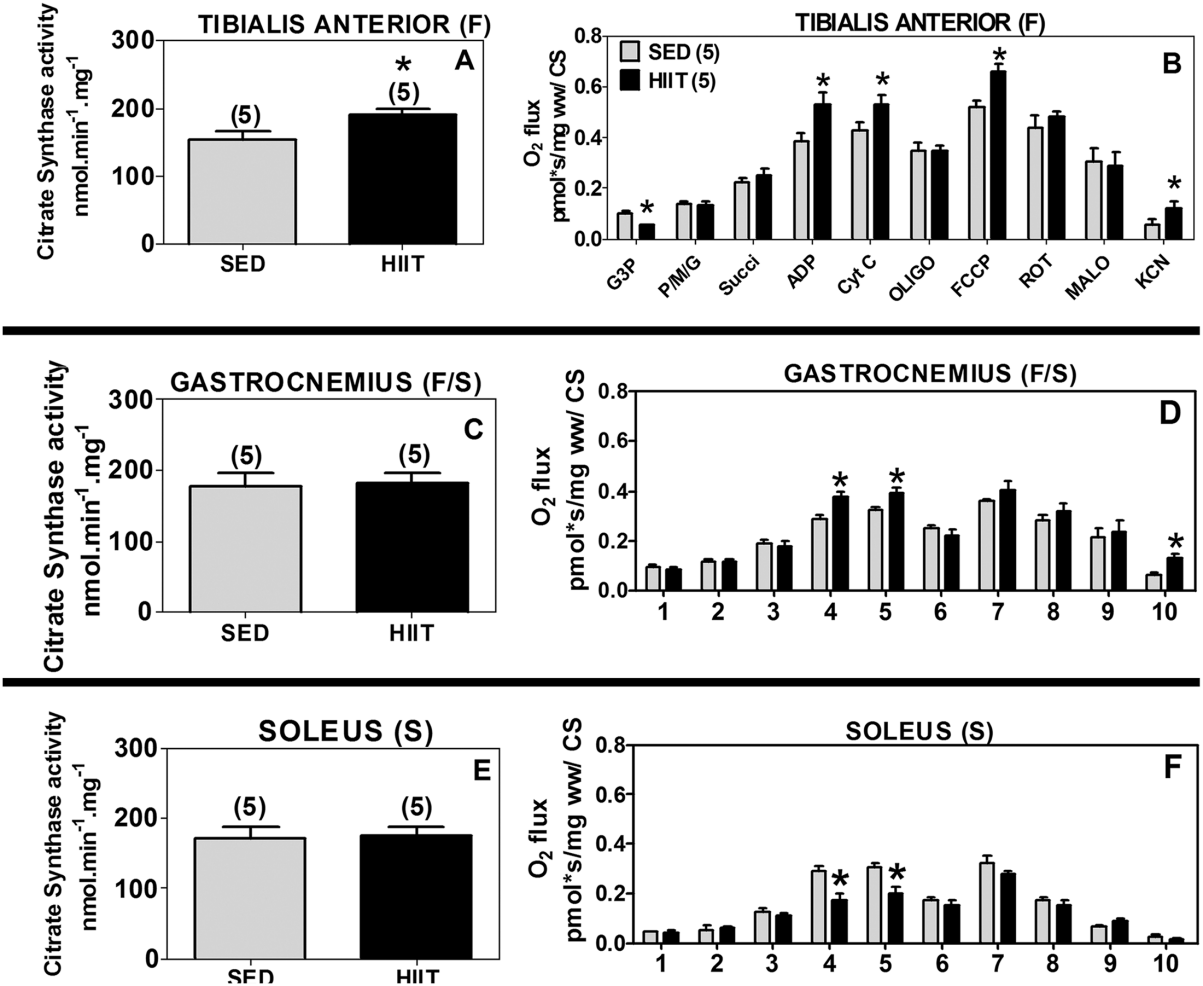
Citrate synthase (CS) activity and oxygen flux in respirometry experiments normalized to CS activity. (A and B) Tibialis anterior. (C and D) Gastrocnemius. (E and F) Soleus. 1, G3P (10 mM); 2, PMG (5;5;10 mM); 3, Succ (10 mM); 4 ADP (2.5 mM); 5, CYT C (10 μM); 6, Oligo (2 μg/ml); 7, FCCP (1 μM); 8, ROT (1.0 μM); 9, MALO (10 mM); 10, KCN (5 mM). Black (HIIT); gray (SED). The data are expressed as the mean ± standard error. *p<0.05, HIIT vs SED. The number of individual experiments is shown in parentheses.

CS activity was measured in the three different muscles as a marker of mitochondrial content. The specific activity of CS varied among the muscle samples from 150 to 200 nmol.min^-1^.mg^-1^ (Fig 3A, 3C and 3E). Despite this variation, the specific activity of CS increased by 1.3-fold in the TA (F) of the HIIT group compared to the SED control group (Fig 3A, p<0.05). The specific activity of CS did not change in the GAST (F/S) and SOL (S) muscles (Fig 3C and 3E). Because CS activity represents mitochondrial content and because the activity levels varied by approximately 30% (Fig 3A, 3C and 3E), all the results were additionally corrected based on CS activity to validate the actual mitochondrial adaptations in the different skeletal muscles to HIIT.

### HIIT induces differential oxygen consumption in distinct skeletal muscles: Data from multiple substrates (glycerol phosphate; pyruvate, malate, and glutamate; and succinate) normalized to citrate synthase

To confirm the results of mitochondrial respiration normalized per milligram of fresh muscle, which could be affected by mitochondrial content, we normalized the mitochondrial respiration results to CS enzyme activity.

In the TA (F), the HIIT protocol was confirmed to decrease the G3P-induced respiration by 43% (p<0.05) and increase the maximum coupled respiration (1.4-fold, p<0.05), maximum uncoupled respiration (1.3-fold, p<0.05) and non-mitochondrial respiration (2.1-fold, p<0.05) compared to the SED group (Fig 3B).

In the GAST (F/S), the HIIT protocol also increased the maximum coupled respiration (1.3-fold, p<0.05) and non-mitochondrial respiration (5.0-fold, p<0.05) compared to the SED group (Fig 3D).

In the SOL (S), the above results validated that the HIIT protocol decreased the maximum coupled respiration by approximately 39% (p<0.05) compared to the SED group (Fig 3F).

### Western blot of OXPHOS content

**Fig 4:**
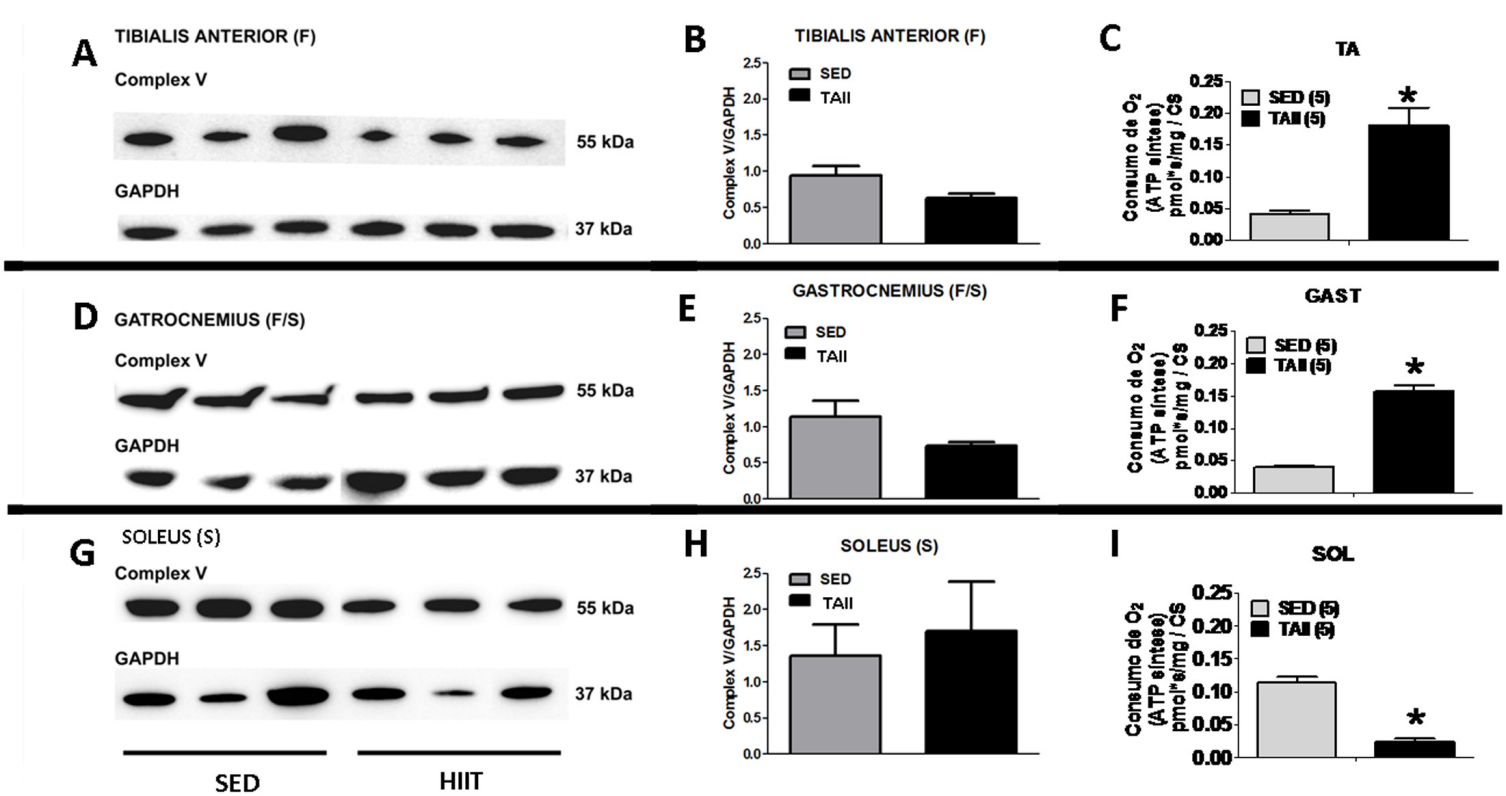
Comparison of complex V content (Western blot) and ATP synthesis capacity (oligomycin-sensitive O_2_ consumption) among the three muscles. Western Blots (A, B and C) for the tibialis anterior, (D, E and F) gastrocnemius, and (G, H and I) soleus. GAPDH was used as the loading control. The data are expressed as the mean ± standard error. *p<0.05, SED vs HIIT. n = 3 for the blot quantification for both the SED and HIIT groups.

The ATP synthesis capacity, as evaluated by the difference in oxygen flux in the presence of ADP + Pi subtracted to the flux of Oligo (a specific inhibitor of FoF1-ATP synthase), revealed that HIIT improved ATP synthesis in the TA and GAST but decreased ATP synthesis in the SOL (for fibers, see Table 2; for mitochondrial content, see Fig 3). These results could be initially interpreted as reflecting different complex V content in each mitochondrion. Thus, the content of the α-subunit of complex V and Oligo-sensitive oxygen flux were compared after HIIT (Fig 4). No differences were observed in complex V content in the HIIT group compared to the SED group in the TA (F), GAST (F/S) or SOL (S) (Fig 4B, 4E and 4H, respectively). Nevertheless, the ATP synthesis-related oxygen flux changed in different ways in the various muscles. ATP synthesis-related oxygen flux increased 4.5-fold and 4.0-fold in TA and GAST fibers, respectively, (Fig 4C and 4F) but decreased by almost 80% in SOL fibers in the HIIT group compared to the SED group (p<0.05) (Fig 4I).

### Oxidation of glycerol phosphate

**Fig 5:**
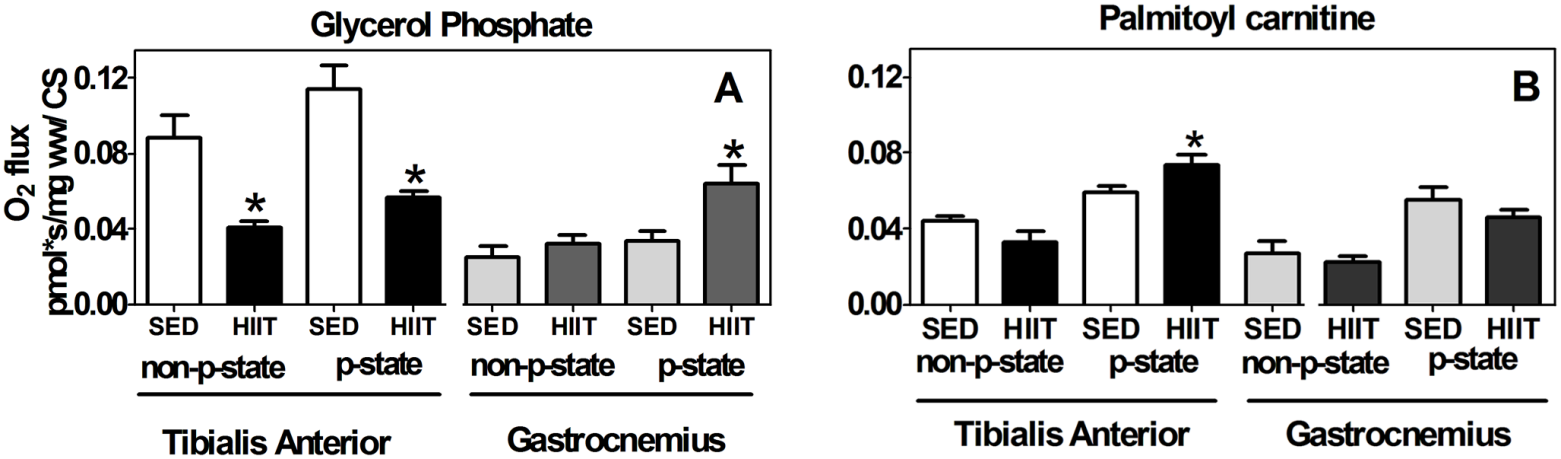
Oxygen flux in mitochondria oxidizing glycerol phosphate (A) in the non-phosphorylated state (non-p-state) and phosphorylated state (p-state) in the tibialis anterior and gastrocnemius, normalized to CS activity. The same experimental protocol was performed using palmitoyl carnitine (B) as a substrate in the tibialis anterior and gastrocnemius with normalization to CS activity. Tibialis Anterior: white (SED) and black (HIIT). Gastrocnemius: light gray (SED) and dark gray (HIIT). The data are expressed as the mean ± standard error. *p<0.05, HIIT vs SED. The number of individual experiments (n) is shown based on 5 rats per group.

To confirm the G3P oxidation results in the multi-substrate condition, we analyzed each substrate separately. When G3P oxidation was normalized to CS activity, we observed a reduction of approximately 55% (p<0.05) in the non-p-state and approximately 51% (p<0.05) in the p-state (Fig 5A). These results are in agreement with those obtained in the multi-substrate condition (Fig 3B). Surprisingly, in the GAST (F/S), HIIT did not affect the oxidation of G3P in the non-p-state, although HIIT stimulated G3P in the p-state by approximately 1.9-fold (p<0.05) (Fig 5A).

### Oxidation of palmitoyl carnitine

Because G3P oxidation decreased after HIIT in the TA but not in the GAST or SOL, we decided to evaluate the effect of HIIT on fatty acid β-oxidation because this pathway sustains respiration in skeletal muscle mitochondria. Therefore, we measured oxygen consumption in TA and GAST muscle fibers using only PALM (Fig 5B) after normalization to CS. Surprisingly, HIIT resulted in a 1.2-fold increase (p<0.05) in the oxygen consumption rate in the p-state of the TA (F). In GAST muscle fibers, the oxygen consumption rate did not change in either the non-p-state or the p-state (Fig 5B).

### H_2_O_2_ production in muscle fibers in the presence of glycerol phosphate and multiple substrates expressed in wet weight (mg) and normalized to citrate synthase

**Fig 6:**
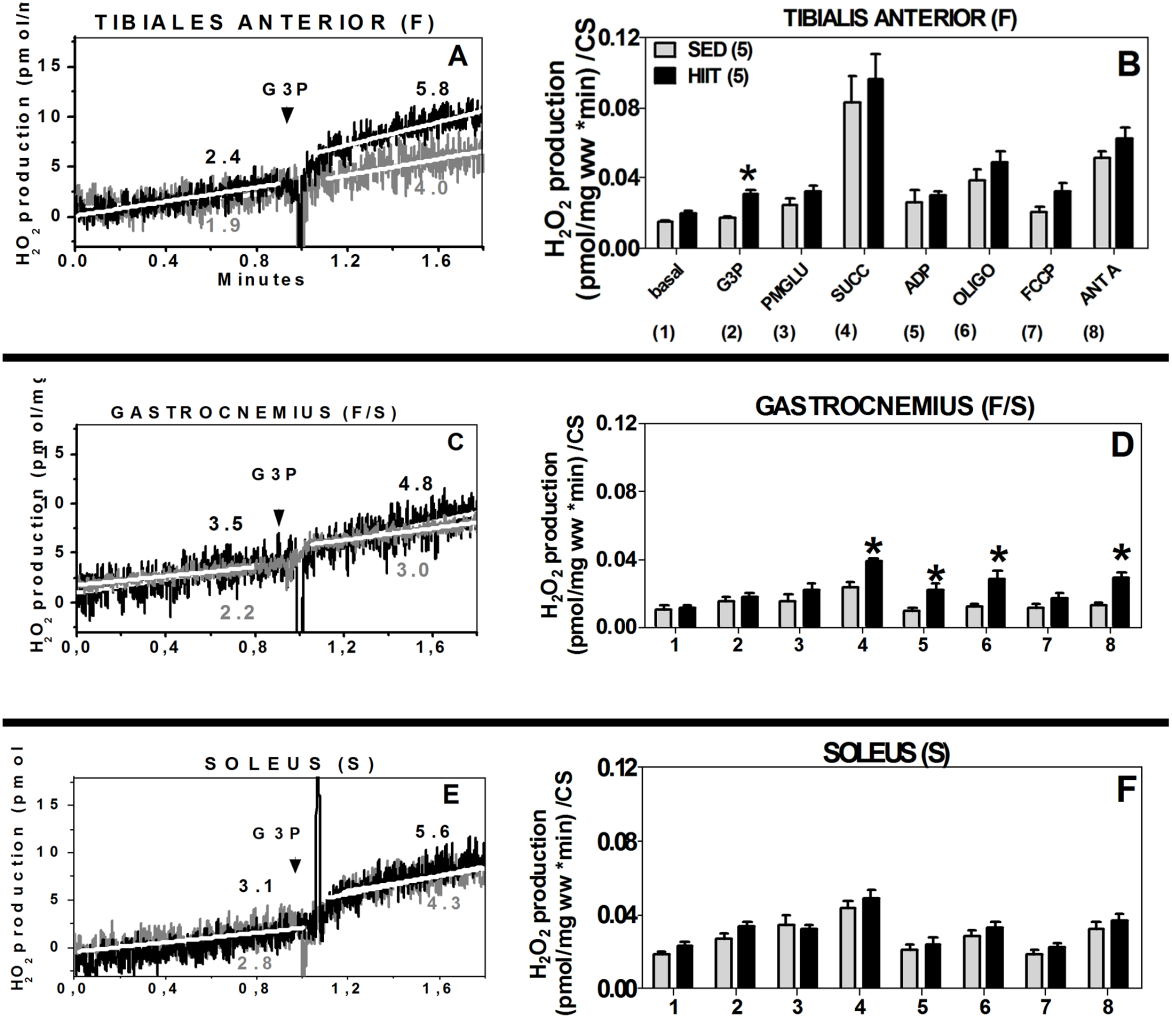
Hydrogen peroxide production using multiple titrations. Tibialis anterior (F) (A and B), gastrocnemius (F/S) (C and D) and soleus (S) (E and F) normalized to wet weight (mg) or citrate synthase. 1, Basal; 2, G3P (10 mM); 3, PMG (5:5:10 mM); 4, Succ (10 mM); 5, ADP (2.5 mM); 6, Oligo (2 μg/ml); 7, FCCP (1 μM); 8, Ant A (5 μM). Black (HIIT); grey (SED). The data are presented as the mean ± standard error. *p<0.05, HIIT vs SED. The number of individual experiments (n) is shown; 5 rats were included in each group.

In the SED group, when G3P/PMG/Succ were added together, the rate of H_2_O_2_ production in the TA was 3.6-fold higher than that in the GAST and 1.9-fold higher than that in the SOL (Fig 6B, 6D and 6F).

TA (F). The HIIT group showed a 1.3-fold increase in H_2_O_2_ production at the site of mGPdH (p<0.05, Fig 6A and 6B). Interestingly, when the data were normalized to CS, H_2_O_2_ generation in the presence of G3P only increased 1.8-fold (p <0.05) in the HIIT group compared to the SED group (Fig 6B).

GAST (F/S). The HIIT group exhibited higher H_2_O_2_ production with the addition of multiple substrates when the data were normalized to CS (Fig 6D). At the site of mGPdH, no significant differences were observed (Fig 6C and 6D). H_2_O_2_ production increased, and there was a 1.6-fold change (p<0.05) in the presence of Succ, which targets complex II. In the presence of ADP, H_2_O_2_ production changed by 1.8-fold (p<0.05), and when OXPHOS was inhibited by Oligo, a 2.2-fold increase was detected (p<0.05). When complex III was inhibited, which results in conditions that favor semiquinone radical formation, a 2.2-fold change (p<0.05) in H_2_O_2_ production was observed (Fig 6D).

SOL (S). When the data were normalized to CS, we did not observe any significant differences in H_2_O_2_ production in the presence of G3P (Fig 6E) or of multiple substrates (Fig 6F).

### H_2_O_2_ production in TA and GAST fibers with glycerol phosphate as a unique substrate

The previous experiments (Fig 6) showed that HIIT enhanced H_2_O_2_ production in the TA and GAST; however, increased H_2_O_2_ generation derived from G3P was observed only in the TA. Thus, we decided to assess whether the activation, inhibition or uncoupling of OXPHOS could modify the rate of electron leakage in the TA and GAST after HIIT. For this assay, G3P was used as a unique substrate.

**Fig 7:**
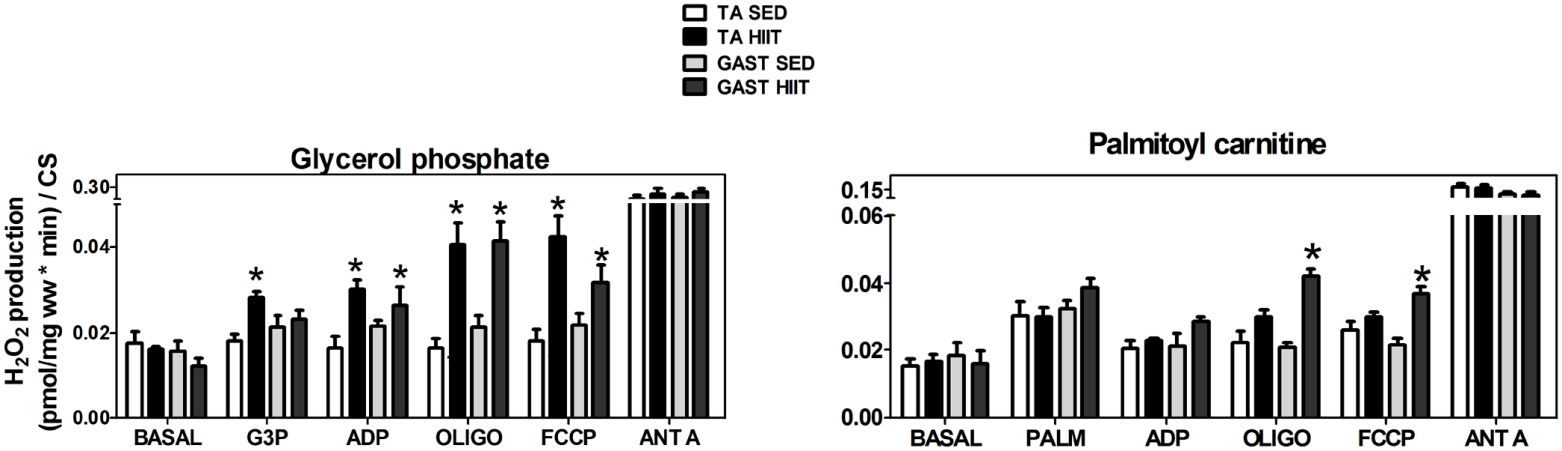
Hydrogen peroxide production by oxidizing glycerol phosphate (A) or palmitoylcarnitine (B) as unique substrates in the tibialis anterior and gastrocnemius after normalization to CS. Tibialis anterior: white, SED; black, HIIT. Gastrocnemius: light gray, SED; dark gray, HIIT. The data are expressed as the mean ± standard error. *p<0.05, HIIT vs SED. The number of individual experiments (n) is shown; 5 rats were included in each group.

TA (F). The HIIT group exhibited higher H_2_O_2_ production compared to the SED group after normalization to CS. H_2_O_2_ generation increased by 2.5-fold at mGPdH sites (p<0.05), by 1.9-fold (p<0.05) in the presence of ADP, by 2.5-fold (p<0.05) in the presence of Oligo, and by 2.3-fold (p<0.05) in the uncoupled state. Blocking complex III, which favors semiquinone radical formation, did not change the maximal H_2_O_2_ levels (Fig 7A).

In another set of experiments, the effect of HIIT on the G3P-mediated reverse flow of electrons to complexes I and II of the ETS in TA muscle fibers was evaluated. We confirmed that HIIT up-regulated the production of H_2_O_2_ by mGPdH 1.3-fold (p<0.05) and by ROT 1.4-fold (p<0.05) compared to the SED group. However, no differences were observed in the reverse flux of electrons in the ETS from complex I or complex II in response to HIIT (Table 3).

**Table 3:** Production of H_2_O_2_ induced by glycerol phosphate as a unique substrate and with inhibitors of complexes I and II in the TA and GAST muscles. SED or trained rats (HIIT), normalized to mg ww/CS.

	Groups	Basal	G3P	ROT	MALO	ANT A
**TA**	**SED (4)**	0.021 ± 0.003	0.024 ± 0.001	0.027 ± 0.003	0.023 ± 0.001	0.146 ± 0.037
	**HIIT (6)**	0.022 ± 0.002	0.029 ± 0.002*	0.036 ± 0.006*	0.026 ± 0.008	0.151 ± 0.050
**Gastro**	**SED (4)**	0.027 ± 0.002	0.040 ± 0.011	0.045 ± 0.008	0.042 ± 0.009	0.134 ± 0.022
	**HIIT (6)**	0.028 ± 0.008	0.037 ± 0.009	0.042 ± 0.010	0.032 ± 0.008	0.164 ± 0.0050

The number of individual experiments is shown in parentheses. The data are expressed as the mean ± standard error.

* p<0.05 vs controls.

GAST (F/S). H_2_O_2_ production increased 2.0-fold when OXPHOS was inhibited with Oligo (p<0.05) and 1.5-fold (p<0.05) in the presence of an uncoupled state in response to FCCP in the HIIT group. As shown in Fig 7A, inhibiting complex III, which favors semiquinone radical formation, did not change the maximal H_2_O_2_ generation. In a complementary experiment, we analyzed the reverse flow of electrons from mGPdH. We did not observe significant differences in any state after HIIT (Table 3).

### H_2_O_2_ production by palmitoyl carnitine as a unique substrate in TA and GAST fibers

Because G3P oxidation was reduced after HIIT in the TA but not the GAST, we evaluated the effect of HIIT on H_2_O_2_ production by fatty acid β-oxidation. Thus, we measured H_2_O_2_ production in TA and GAST muscle fibers using PALM as a unique substrate (Fig 7A).

TA (F). We did not observe any differences using PALM as a substrate after normalization to CS (Fig 7B, white and black columns). In a complementary experiment, we analyzed the reverse flow of electrons from PALM and did not observe any significant differences after HIIT training in any of the H_2_O_2_ production states (Table 4).

**Table 4:** Production of H_2_O_2_ induced by PALM as a unique substrate and with inhibitors of complexes I and II in TA and GAST muscles. SED or trained rats (HIIT), normalized to mg ww/CS.

	Groups	Basal	PALM	ROT	MALO	ANT A
**TA**	**SED (4)**	0.015 ± 0.003	0.028 ± 0.001	0.024 ± 0.001	0.019 ± 0.001	0.043 ± 0.007
	**HIIT (6)**	0.019 ± 0.002	0.026 ± 0.004	0.028 ± 0.004	0.026 ± 0.004	0.045 ± 0.002
**Gastro**	**SED (4)**	0.013 ± 0.002	0.033 ± 0.005	0.022 ± 0.004	0.023 ± 0.004	0.039 ± 0.007
	**HIIT (6)**	0.018 ± 0.005	0.032 ± 0.007	0.033 ± 0.006	0.029 ± 0.003	0.043 ± 0.013

The number of individual experiments is shown in parentheses. The data are expressed as the mean ± standard error.

GAST (F/S). In the HIIT group, H_2_O_2_ production normalized to CS increased when OXPHOS was inhibited with Oligo (2.1-fold, p<0.05) and in the uncoupled state elicited by FCCP (1.7-fold, p<0.05). Inhibiting complex III with ANT A did not affect the maximal H_2_O_2_ generation (Fig 7B, light gray or dark gray columns). In a complementary experiment, we analyzed the reverse flow of electrons from PALM and did not observe significant differences after HIIT for any of the H_2_O_2_ production states (Table 4).

### Electron leakage ratio using G3P or multiple substrates (G3P/PMG/Succ)

**Fig 8:**
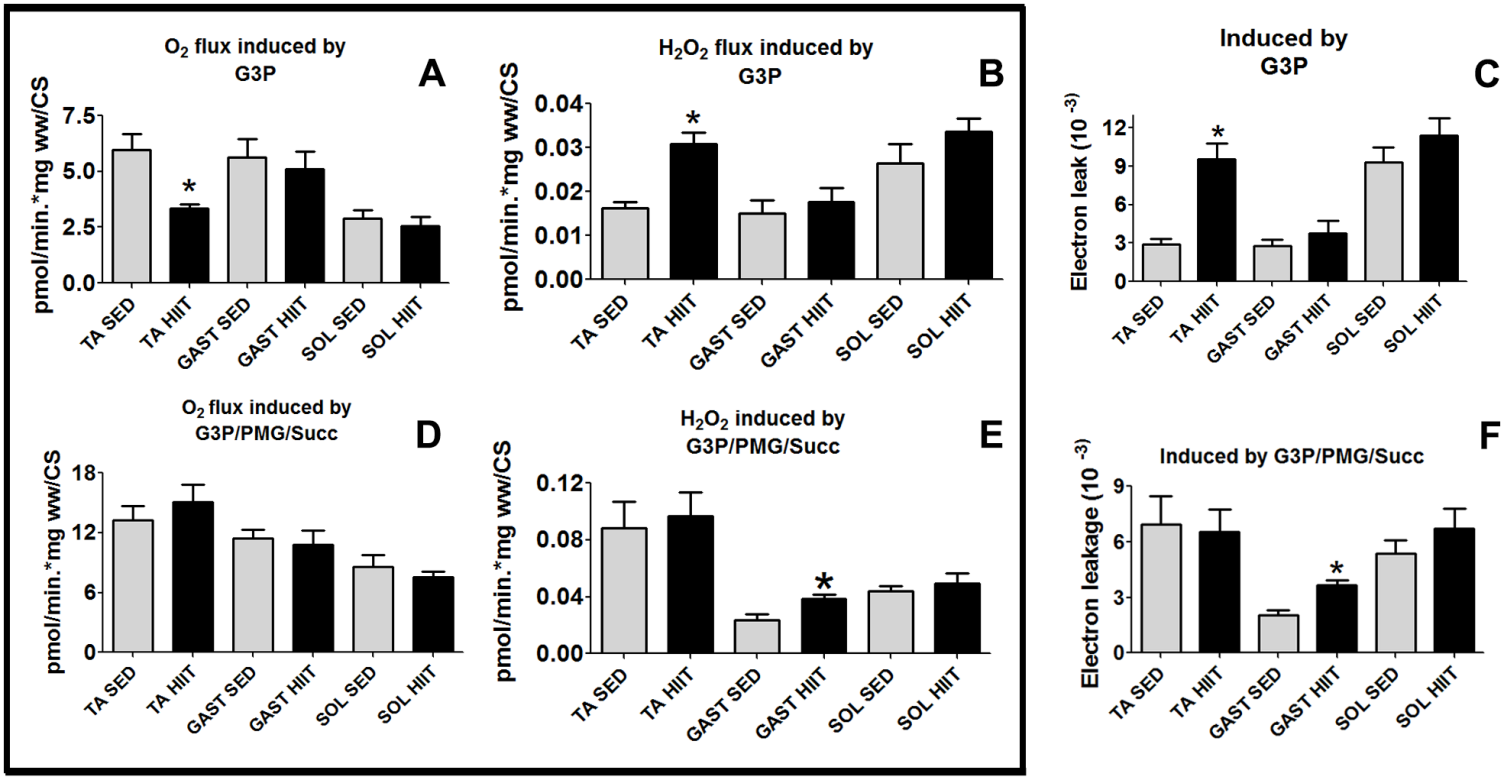
O_2_ flux normalized to CS, H_2_O_2_ production normalized to CS, and electron leakage in the presence of G3P or multiple substrates (G3P/PMG/Succ) for the three muscles. (A) O_2_ flux. (B) H_2_O_2_ production. (C) Electron leakage from oxidizing G3P. (D) O_2_ flux. (E) H_2_O_2_ production. (F) Electron leakage from oxidizing multiple substrates (G3P/PMG/Succ). The data are expressed as the mean ± standard error. *p<0.05, HIIT vs controls. The number of individual experiments (n) is shown based on 5 rats per group. Black (HIIT); white (SED).

We compared the three skeletal muscle fiber types to evaluate the relative contribution of G3P/mGPdH and multi-substrate systems to electron leakage in the presence of G3P/PMG/Succ after HIIT (Fig 8). Surprisingly, despite the fact that the fibers showed different rates of H_2_O_2_ production and mitochondrial respiration, the highest electron leakage ratio was observed for all the fibers when G3P was used as a unique substrate.

TA fibers in the HIIT group showed the highest difference in the electron leakage ratio with G3P (Fig 8C). The O_2_ flux by G3P was decreased by 44% (Fig 8A); nevertheless, H_2_O_2_ production increased by 1.9-fold (p<0.05) (Fig 8B). Accordingly, after HIIT, H_2_O_2_ production was approximately 3.3-fold higher compared to that in the SED group (p<0.05) (Fig 8C).

In the GAST fibers of the HIIT group, the O_2_ flux by G3P/PMG/Succ did not change (Fig 7D), but H_2_O_2_ generation increased 1.6-fold (p<0.05) (Fig 8E). Thus, the electron leakage ratio increased by 1.8-fold (p<0.05) relative to that in the SED group (Fig 8F).

In the SOL fibers, there were no changes in O_2_ flux (Fig 8D) or H_2_O_2_ production (Fig 8E) by G3P/PMG/Succ; therefore, electron leakage in response to G3P/PMG/Succ did not differ between the HIIT and SED groups (Fig 8F).

## Discussion

HIIT has been widely and increasingly discussed because it is metabolically similar to aerobic training but requires much less time spent on training activities to perform the same work. We confirmed that HIIT results in higher maximum swimming duration, lower body weight and lower indexes of adiposity (measured by visceral fat content and visceral fat percentage) [3,15]. Similar to muscle adaptations after endurance training, some mechanisms have been proposed by other authors to explain these features of HIIT, such as a higher content of PGC-1α, a higher amount of GLUT_4_ in the muscle membrane, a higher activity of CS and a higher activity of AMPK [3, 11, 30, 31] for the epitrochlearis muscle. Because it is expected that endurance training induces adaptations in OXPHOS and antioxidant enzyme capacities, it would be also expected that exercise may affect the total electron leakage of skeletal muscle. However, after HIIT, mitochondrial oxygen and H_2_O_2_ flux have not been simultaneously measured in three muscles used in swimming activities with distinct twitch types.

We show here for the first time that HIIT uses multiple substrate feeders of the ETS to modulate mitochondrial respiratory physiology. HIIT increased OXPHOS respiration in TA (F) and GAST (F/S) skeletal muscle fibers but decreased OXPHOS respiration in SOL (S) fibers. The apparent paradox of decreased OXPHOS respiration in SOL fibers during swimming HIIT could be the result of differences in muscle activation, as has been observed in previous electromyography studies conducted in rats. The TA (F) is the most recruited muscle in swimming training in rats. The SOL and GAST are used 12% and 23% less, respectively, in swimming compared to running [32]. Thus, in swimming training, the decreased OXPHOS respiration in the SOL could represent a transition in muscle adaptation from running to swimming. In addition, the TA and GAST showed decreased glycogen levels of 75% and 50%, respectively, during swimming, demonstrating that these fibers are active [22, 23]. In accordance with the electromyography data, glycogen levels did not change in the SOL [32]. In addition, blood flow to the SOL decreases in swimming rats [33]. In other data from hindlimb unweighting experiments, the SOL lost tetanic contraction in about 60% of cases [34]. It is possible that the decreased oxygen delivery to mitochondria could reduce its capacity to produce ATP, thus decreasing the formation of superoxide anions (O^**• -**^) or decreasing the ability of the mitochondria to use substrates such as G3P, pyruvate, malate, glutamate and Succ in oxygen flux.

Here, HIIT-trained rats had higher rates of ATP synthesis in the TA (F) and GAST (F/S) compared to SED controls (Fig 2B and 2D). These results may indicate that the gain in performance (endurance time, compared to the SED group) after HIIT correlates with the gain in OXPHOS capacity and ATP turnover. Similar results have been observed in the vastus lateralis (fast/slow twitch) of trained human athletes [35], trained human non-athletes [36] and trained rats [37]; furthermore, the maximal respiratory capacity has been shown to be higher for glycolytic muscles, a feature that seems to be related to the high expression of PGC-1α protein and mRNA. In the present study, a different consequence of training was observed in Fig 3. It could be hypothesized that there would be no difference in respiration normalized to CS after HIIT because TA fibers showed an approximate 30% increase in CS activity. However, the respiration rates stimulated by ADP or FCCP were even higher than expected based on mitochondrial biogenesis. Thus, different regulatory mechanisms may be involved in the regulation of OXPHOS after HIIT.

The mGPdH-supported respiration using multi-substrate titrations was lower in the HIIT-trained rats. This result is in agreement with the previously reported low activity levels of this enzyme [15]. Hoshino et al. [37] demonstrated that HIIT on a treadmill induced higher amounts of palmitate oxidation in both subsarcolemmal (SS) and intermyofibrillar (IMF) mitochondria isolated from hindlimb muscles. Our results for the TA are in agreement, as the TA promotes higher mitochondrial respiration of PALM in the phosphorylated state (Fig 5A). Nevertheless, the respiration changed in the GAST (Fig 5B). This difference might be explained by the differences in the HIIT protocols (treadmill or swimming), but further studies comparing HIIT protocols are needed to clarify this point in detail.

H_2_O_2_ production using multiple substrates (G3P/PMG/Succ) was higher in fast twitch muscle fibers (TA) than in fast/slow twitch (GAST) or slow twitch (SOL) fibers (Fig 6). These results correspond with the use of pyruvate or malate plus glutamate as substrates for complex I. The use of Succ alone as a substrate (complex II) resulted in detectable differences between the white GAST twitches (WG, primarily type IIB twitches) and the red GAST (RG, type IIA) or SOL (type I) twitches [24].

After HIIT, the TA produced more H_2_O_2_ than the GAST or SOL muscle twitches (Fig 6B, 6E and 6H, respectively), despite the fact that the TA has decreased mGPdH activity. These data suggest that HIIT specifically modulates the redox state and/or the complex shuttle and the ETS of skeletal muscle mitochondria. It has been demonstrated that the total antioxidant capacity and lipid peroxidation of the TA is maintained in HIIT-trained rats compared to SED rats [15]. Moreover, a positive correlation between mGPdH activity levels and H_2_O_2_ production among different rat tissues has been identified [38]. However, there are currently no studies in which the effects of the HIIT protocol on different muscle fiber preparations accurately mimic the effects of HIIT in vivo. One possible explanation for the enhanced H_2_O_2_ production and low activity levels of mGPdH after HIIT is that the interaction between different complexes of the ETS in TA fibers could alter the reverse electron flux. However, we could not detect any changes in reverse flux (Table 3). Thus, it is possible that the HIIT protocol and low oxygen delivery altered COX and supercomplex assembly factor I (SCAFI) peptide, which are involved in the assembly of supercomplexes, including those that contain mGPdH. This would alter the partitioning of electrons in the mGPdH coenzyme Q-binding pocket in TA skeletal muscle after HIIT [39, 40], causing mGPdH to produce superoxides on either side of the mitochondrial membrane in approximately equal amounts, thereby diverting more superoxides toward the outer side of the inner mitochondrial membrane [38]. Further studies to evaluate the interaction of ETS and mGPdH in greater detail and at higher resolution are necessary to verify these predictions.

There was an increased rate of H_2_O_2_ production by substrates of complex I (PMG) and II (Succ) in GAST (F/S) fibers. These results corroborated the increased ROS production in rats after endurance training using the same substrates in vastus lateralis (mixed muscle) [41].

We observed for the first time that HIIT is associated with electron leakage. Impressively, electron leakage at mGPdH sites was higher only in the TA (Fig 7C). The classical adaptations to endurance training suggest that the intracellular responses to ROS production are required for the normal remodeling that occurs in skeletal muscle [42]. Chronic endurance training reduces oxidative stress and thereby confers protection by increasing antioxidant defenses and maintaining redox homeostasis in the ETS [42, 43]. Curiously, Daussin (20) observed that endurance training induced activation of mitochondrial respiration, although the total H_2_O_2_ generation was unchanged; thus, they concluded that electron leakage decreased and that the mRNA expression of genes involved in the antioxidant system increased. In contrast, Isner‐Horobeti et al. [41] demonstrated that electron leakage increased in response to Succ in vastus lateralis (mixed muscle) after endurance training in rats, corroborating our results for the GAST (Fig 8F). However, despite these data, the electron leakage at these sites increased in our study (Fig 8), and it was expected that these points in the ETS in which H_2_O_2_ is produced were important for mediating intracellular ROS production to affect metabolic signaling. In fact, intracellular ROS production is necessary for activating AMPK phosphorylation to control glucose and PGC-1α homeostasis [44].

## Conclusion

HIIT promoted specific alterations in mitochondrial respiration to increase OXPHOS respiration in the TA and GAST and decrease this respiration in the SOL; furthermore, the production of H_2_O_2_ was enhanced in the TA and GAST but was not altered in the SOL. Specifically, electron leakage was higher in the TA with G3P as the substrate and in the GAST with G3P/PMG/Succ but did not change in the SOL after HIIT.
